# Concurrent *KRAS* p.G12C mutation and *ANK3::RET* fusion in a patient with metastatic colorectal cancer: a case report

**DOI:** 10.1186/s13000-024-01478-1

**Published:** 2024-03-27

**Authors:** Tillmann Bedau, Carina Heydt, Udo Siebolts, Thomas Zander, Max Kraemer, Heike Loeser, Reinhard Buettner, Alexander Quaas

**Affiliations:** 1 Department of Pathology, University Hospital Cologne, Cologne, Germany; 2 Department I of Internal Medicine, Center for Integrated Oncology (CIO), University of Cologne, Cologne, Germany; 3 Institut für Pathologie Köln-Deutz, Cologne, Germany

**Keywords:** *KRAS* p.G12C mutation, *ANK3::RET* fusion., Metastatic colorectal cancer, Molecular diagnostics, Targeted therapy, Precision oncology, Genetic profiling, RET kinase inhibitors, KRAS inhibitors, Novel therapeutic strategies

## Abstract

**Background:**

Colorectal cancer (CRC) frequently involves mutations in the *KRAS* gene, impacting therapeutic strategies and prognosis. The occurrence of *KRAS* mutations typically precludes the presence of *RET* fusions, with current medical literature suggesting a mutual exclusivity between these two genetic alterations. We present a unique case that challenges this notion.

**Case Presentation:**

An 85-year-old female with metastatic CRC was found to have a combination of genetic anomalies that is to the best of our knowledge not yet described in the medical literature: a *KRAS* p.G12C mutation, associated with oncogenesis and treatment resistance, and an *ANK3::RET* fusion, an infrequent but targetable mutation in CRC. This molecular profile was uncovered through comprehensive genomic sequencing after the patient experienced metachronous tumor dissemination. The presence of both genetic events complicates the treatment approach.

**Conclusions:**

The identification of both a *KRAS* p.G12C mutation and an *ANK3::RET* fusion in the same CRC patient adds a new layer to the oncogenic landscape and treatment considerations for CRC. It highlights the intricate decision-making required in the era of precision medicine, where targeted therapies must be carefully chosen and potentially combined to combat complex genetic profiles. The case emphasizes the urgency of investigating the clinical effects of concurrent or sequential use of *KRAS* p.G12C and *RET* inhibitors to inform future therapeutic guidelines and improve patient outcomes in similar cases.

**Supplementary Information:**

The online version contains supplementary material available at 10.1186/s13000-024-01478-1.

## Background

The *RET* proto-oncogene encodes a receptor tyrosine kinase, which is crucial in cell signaling. Abnormal activation of its signaling functionality has been associated with several malignancies and can occur via activating mutations (as in multiple endocrine neoplasia type 2, MEN2) or via fusion with other proteins leading to ligand-independent *RET* signaling [1, 2]. *RET* fusions are predominantly found in 5–10% of patients with papillary thyroid carcinoma (PTC) and 1–2% of patients with non-small-cell lung cancer (NSCLC) [3]. In colorectal cancer (CRC), only a small fraction of tumors (< 1%) harbors a *RET* fusion of *RET* exon 11 or 12 [4–7] with the most common being *NCOA4::RET* and *CCDC6::RET* fusions [8]. RET fusions, characterized by the juxtaposition of the RET kinase domain with dimerization domains from various partners, typically lead to ligand-independent dimerization, constitutive kinase activation and oncogenic signaling through pathways such as MAPK, PI3K/AKT, and JAK/STAT, promoting cell proliferation and survival [2, 9]. The discovery of RET fusions across a spectrum of cancers has underscored their role as actionable targets for kinase inhibitor therapy, with their presence often indicating sensitivity to specific RET inhibitors. The detection of oncogenic fusions has evolved significantly with advances in molecular diagnostics. Initially identified through fluorescence in situ hybridization (FISH), the advent of next-generation sequencing (NGS) technologies has greatly enhanced our ability to detect these fusions with high sensitivity and specificity. DNA/RNA-based NGS, in particular, has become a cornerstone in the identification of RET fusions, allowing for the comprehensive profiling of cancer genomes and the detection of fusions across a wide range of known and novel partner genes. This approach, complemented by confirmatory assays such as FISH for visual confirmation of chromosomal rearrangements, enables a robust framework for the molecular characterization of tumors and the identification of potential therapeutic targets [10].

The members of the RAS family of proteins encoded by *KRAS*, *NRAS*, and *HRAS* act as GTPases at the cytosolic side of the plasma membrane. Upon activation of transmembrane receptor tyrosine kinases, they transmit mostly pro-proliferative signals to the cell [11]. Aberrant RAS signaling is a key oncogenic mechanism, reflected by *KRAS* being one of the most commonly mutated oncogenes in human cancer [12]. In CRC, *KRAS* is mutated in around 40% of cases [13]. The global median prevalence of the *KRAS* p.G12C mutation in CRC is 3.1% [14].

In the molecular landscape of CRC, a pivotal aspect is the generally mutually exclusive nature of *KRAS* mutations and oncogenic fusions like *RET* [1, 4]. *KRAS* mutations, usually thought of as initial drivers in tumorigenesis, lead to persistent activation of signaling pathways, making the cell less reliant on external growth signals. This mechanistic pathway typically negates the need for additional oncogenic drivers, such as *RET* fusions. Although the co-occurrence of *KRAS* mutations and *RET* fusions in CRC is historically considered rare and literature to date consistently reports *RET* fusions exclusively in the context of *KRAS* wild-type tumors, genetic combinations, including variances of unknown significance like the fusion reported here, can indeed occur, suggesting that the interplay of genetic alterations is more complex than previously understood. This established understanding forms the backdrop against which our case stands out, presenting a unique combination of a *KRAS* p.G12C mutation and an uncommon *ANK3::RET* fusion.

## Case presentation

**Fig. 1:**
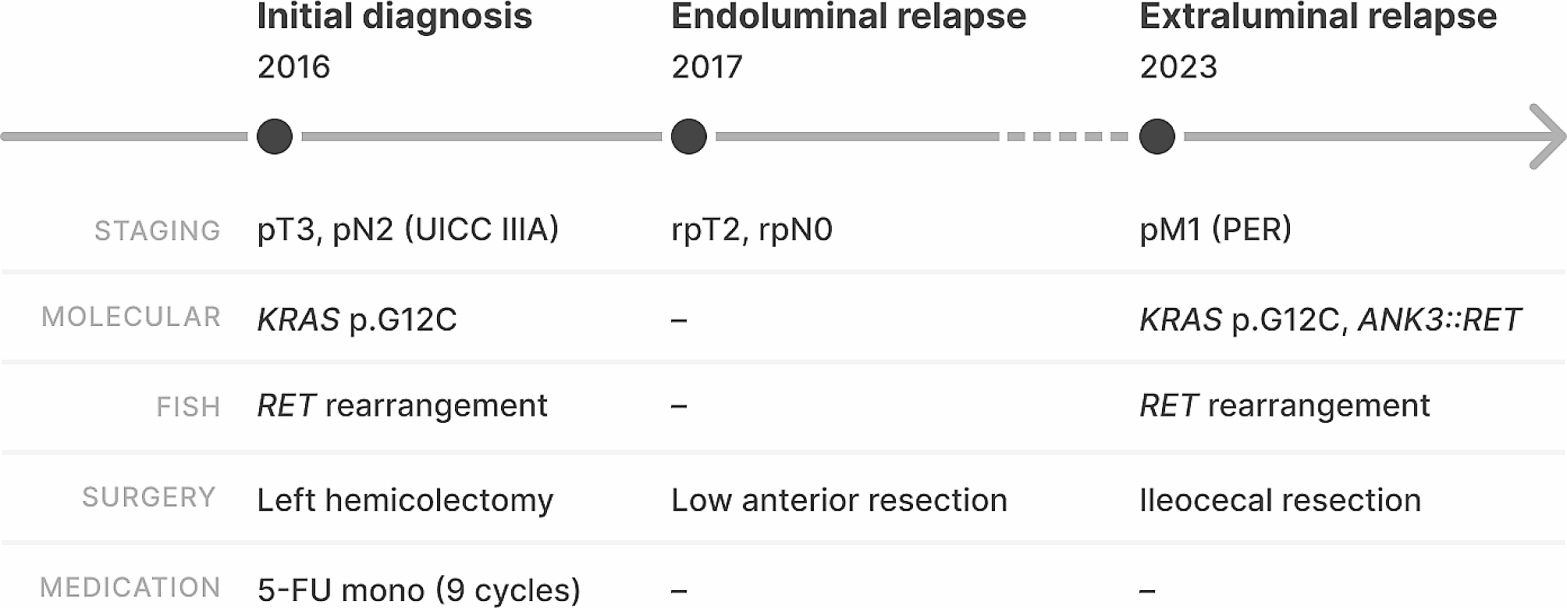
Timeline of the patient’s clinical course and treatments. The timeline shows key events from the initial diagnosis of colorectal cancer (CRC) in 2016 to the current metastatic disease stage. Staging information is provided according to the Union for International Cancer Control (UICC) classification system. Relevant molecular findings as well as corresponding interventions, including surgery and medication, are detailed for each time point

**Fig. 2:**
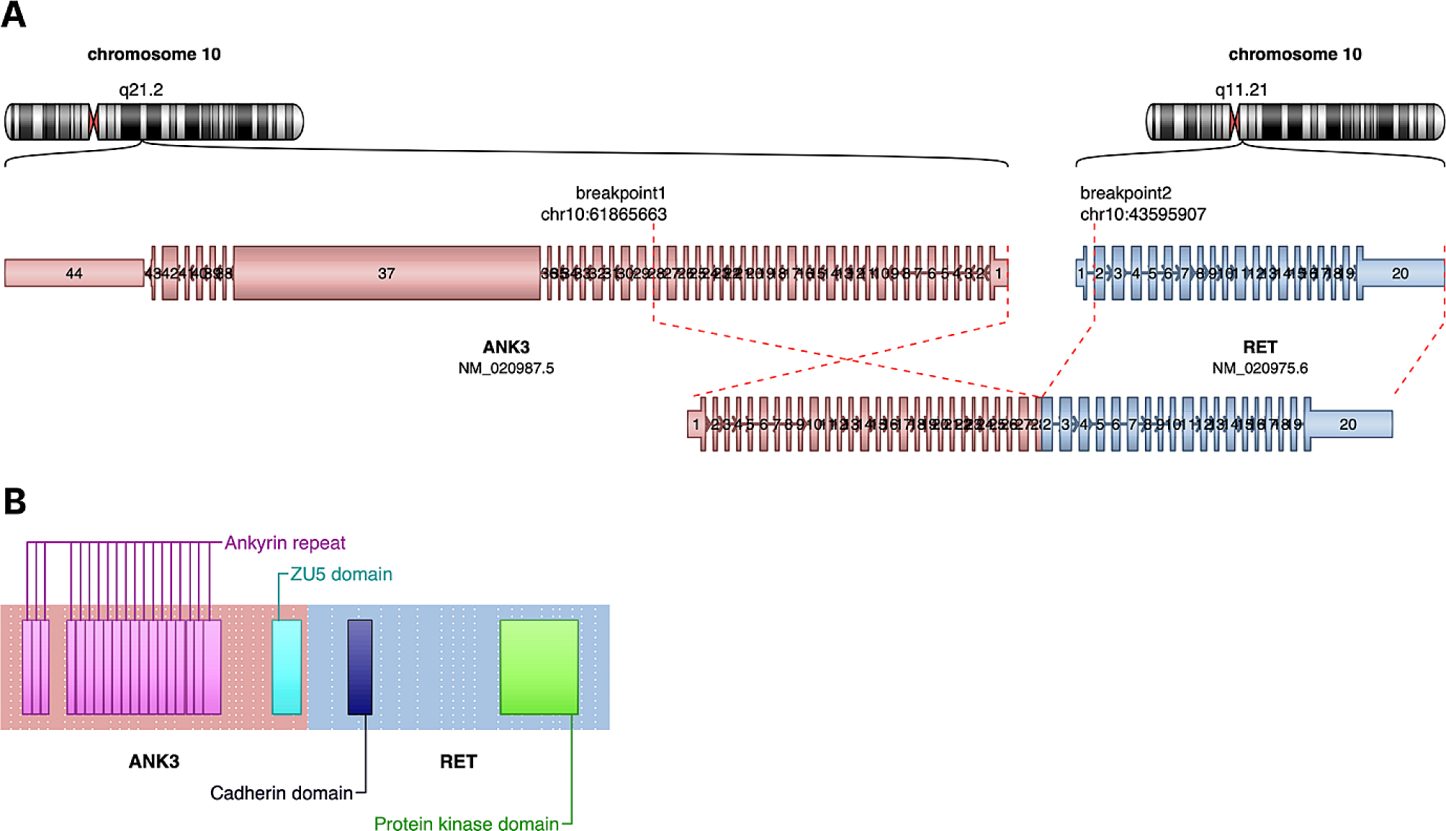
Schematic representation of the *ANK3::RET* fusion event. (**A**) Chromosomal locations of the breakpoints within chromosome 10 leading to the fusion of the genes *ANK3* and *RET* genes. Breakpoint 1 (chr10:61865663; reference genome: hg19) occurs after exon 28 of *ANK3*, and breakpoint 2 (chr10:43595907) before exon 2 of *RET*. (**B**) Domain structure of the fusion protein, with the *ANK3* gene contributing its ankyrin repeats and ZU5 domain, fused to the *RET* gene with intact cadherin and protein kinase domains. The resulting chimeric protein retains key functional domains from both original proteins. Both visualizations were created using the gene fusion detection tool Arriba [15]

**Fig. 3:**
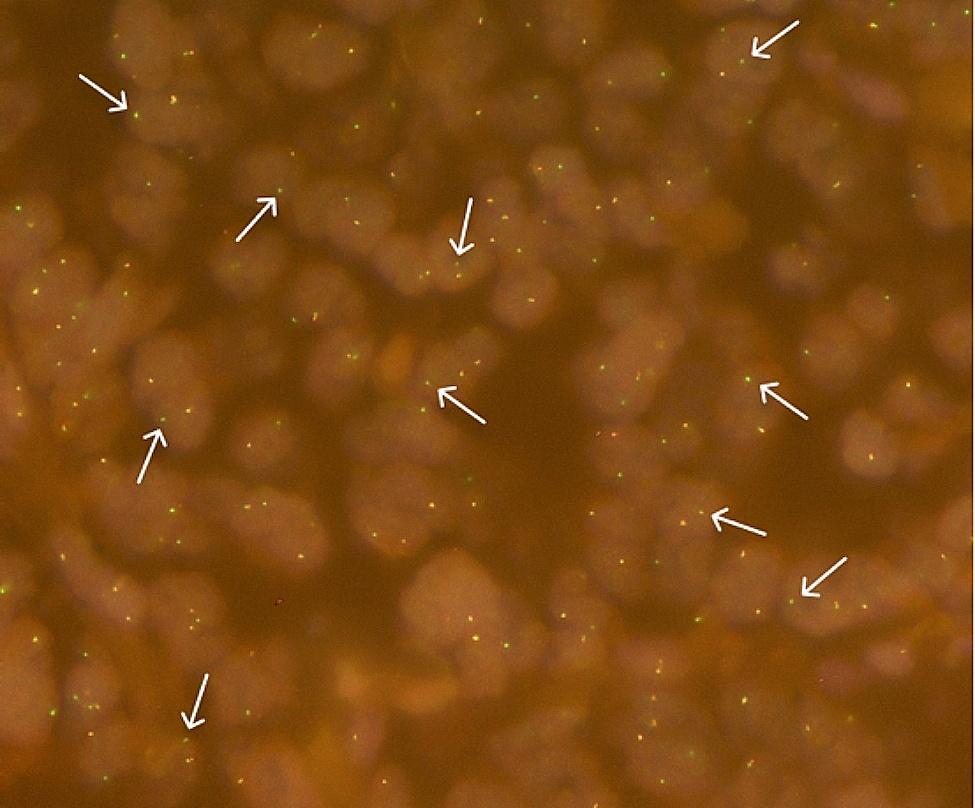
Fluorescence in situ hybridization (FISH) analysis for *RET* gene rearrangement. The tissue sample was hybridized with a break-apart probe for *RET*, where the separation of red and green signals indicates a translocation involving the *RET* locus. An extra green signal pattern was primarily observed in 93% of tumor cells (white arrows), break-apart signals were observed in 2% of tumor cells. Original magnification, x63

An 85-year-old female patient was admitted to our outpatient department due to newly diagnosed colorectal peritoneal metastasis. Prior evaluation of progressive fatigue and weight loss in a different hospital revealed a suspicious 4 × 7 cm tumor mass in the upper abdomen. Subsequent extended ileocecal resection and pathological evaluation led to the diagnosis of extraluminal CRC relapse with tumor dissemination from the mesentery extending into the terminal ileum and cecum. The patient had initially been diagnosed with adenocarcinoma located at the junction of the descending and sigmoid colon in April 2016 (pT3, pN2 (4/14), V0, L0; G2; UICC IIIA). The tumor was microsatellite stable (MSS) and harbored a *KRAS* G12C mutation (*NRAS/BRAF* wild type). Initial therapy consisted of left hemicolectomy followed by 9 cycles of adjuvant 5-FU monotherapy. In 2017, the patient had an endoluminal relapse (rpT2, rpN0 (0/2), L0, V0; G2), which was treated with low anterior resection (clinical timeline illustrated in Fig. 1). The patient has a notable family history of CRC, including diagnoses in her sister, mother as well as maternal uncle, aunt, and grandfather. Comorbidities include diabetes mellitus, atrial fibrillation, chronic kidney disease, and COPD. Now, upon metachronous dissemination of the tumor, a comprehensive TruSight Oncology 500 (TSO500) assay (Illumina, San Diego, USA), performed on a tumor specimen from the recent ileocecal resection, confirmed the initial *KRAS* p.G12C mutation and additionally identified an *ANK3*(Ex.28)::*RET*(Ex.2) fusion with breakpoints located at position chr10:61865663 and chr10:43595905, respectively (Fig. 2). This molecular event combination is particularly unusual, given the existing understanding that known *RET* fusions are typically exclusive to *RAS* wild-type tumors in CRC or other tumors like NSCLC. Furthermore, next-generation sequencing (NGS) revealed an activating mutation in *IDH1* (p.R132C), a truncating mutation in *APC* (Table 1), and several other gene mutations of unknown significance (Supplementary Table 1). The tumor exhibited a high mutational burden (10.2 mut/Mb). No gene amplifications or other fusions were detected. The *ANK3*(Ex.28)::*RET*(Ex.2) fusion was confirmed using the FusionPlex Lung Kit (ArcherDX, Boulder, USA) and by fluorescence in situ hybridization (FISH, Fig. 3). A retrospective analysis of the primary tumor material from 2016 and a tumor-infiltrated lymph node from the same period revealed the presence of *RET* gene rearrangement. This finding indicates that the RET fusion was an early event in the disease’s progression (Supplementary Fig. 1).

**Table 1:** Gene variants with functional and/or clinical significance detected by the TSO500 assay

Gene	Allele frequency	Coverage	Variant (p.)	Variant (c.)	Exon	Information
*APC*	67.25	858	NP_000029.2: p.(His1490IlefsTer17)	NM_000038.5: c.4468del	16/16	Truncated protein, likely loss-of-function (OncoKB), pathogenic (ClinVar)
*IDH1*	23.88	934	NP_005887.2: p.(Arg132Cys)	NM_005896.3: c.394 C > T	4/10	Activating (Jax-CKB)
*KRAS*	51.62	494	NP_203524.1: p.(Gly12Cys)	NM_033360.3: c.34G > T	2/6	Activating (Jax-CKB)

## Discussion and conclusions

In this report, we describe a fusion of *RET* exon 2 in a CRC patient, with exon 28 of Ankyrin-3, encoded by *ANK3*, as the fusion partner. The ankyrin family of proteins is involved in linking membrane proteins to the cytoskeleton. To our knowledge, there are only three reports of *ANK3::RET* fusions in the medical literature, all having been discovered in NSCLC patients, with the fusion event affecting *RET* exon 12 in each case [16–18]. The biological significance of these fusion events is unknown. It is noteworthy that the *RET* breakpoint identified in our case is located in exon 2. This positioning retains the entire protein structure, comprising the large extracellular domain, the transmembrane domain, and the intracellular kinase domain. Contrastingly, most previously described *RET* fusions feature breakpoints in exon 11 or 12. In these instances, only the cytoplasmic part of the protein, which contains the kinase domain, is preserved [2]. Such a difference in the breakpoint location could imply distinct functional implications for the *ANK3::RET* fusion detected here compared to other known RET fusions.

Our case report delineates an exceptional occurrence of concurrent *KRAS* G12C mutation and *RET* fusion, a combination challenging the prevailing notion of mutual exclusivity between *RAS* mutations and *RET* fusions in CRC. This dual molecular alteration could suggest either a novel synergistic or a parallel oncogenic mechanism. It raises the question of whether the *KRAS* mutation and *RET* fusion are functionally independent with the *RET* fusion being just a random bystander event or whether there is a potential cross-talk or compensatory mechanism between these pathways in this patient’s tumor.

The therapeutic decision-making is far from straightforward in this case. Parallel to the consideration of RET inhibition, recent advancements in targeting *KRAS* mutations present an additional therapeutic dimension. Historically labeled as “undruggable”, the landscape of targeting *KRAS* mutations has evolved with the advent of novel *KRAS* p.G12C small molecule inhibitors like Sotorasib and Adagrasib. In CRC, these inhibitors have shown promising results in combination with epidermal growth factor receptor (EGFR) inhibition (Cetuximab and Panitumumab) to account for potential treatment-induced resistance mediated by upstream reactivation of the EGFR pathway [19, 20]. In fact, the combination of either of these drugs (*KRAS* p.G12C inhibitor + anti-EGFR monoclonal antibody) is now recommended for CRC with level 2 evidence in OncoKB.

Additionally, the therapeutic potential of RET inhibition in this case warrants consideration. OncoKB [21, 22] currently lists selective RET kinase inhibitors Pralsetinib and Selpercatinib as targeted therapy options for *RET* fusion-positive NSCLC and thyroid cancer with level 1 evidence of clinical actionability based on results of the ARROW (NCT03037385) [23] and LIBRETTO-001 (NCT03157128) [1, 24] trials, respectively. In the case of Selpercatinib, there is also level 1 evidence for all solid tumors apart from NSCLC and thyroid cancer with an objective response rate of 43.9% in a phase 1/2 basket trial [3]. However, no patient enrolled in these clinical trials carried an *ANKR3::RET* fusion and, importantly, the presence of other oncogenic drivers such as *KRAS* mutations was reason for exclusion. Consistent with this data, the above-mentioned NSCLC patients harboring an *ANKR3:RET* fusion [17, 18] were treated with Pralsetinib, resulting in a documented tumor response.

In light of these findings, we are confronted with a therapeutic conundrum. The *RET* fusion, typically a promising target for selective inhibitors like Pralsetinib and Selpercatinib, is complicated by the concurrent presence of a *KRAS* p.G12C mutation. This mutation acts downstream in the cell signaling pathways and could potentially override the effects of inhibiting the RET fusion, which operates at an earlier, or upstream, point in these pathways. The critical question arises: Should the therapy focus on the upstream *RET* fusion using available inhibitors, or should it target the downstream *KRAS* p.G12C mutation, for which the new inhibitors are showing promise? The possibility of using both approaches at the same time also presents itself, yet this strategy is uncharted in clinical practice, with insufficient evidence to predict outcomes.

In conclusion, this case encapsulates the challenges faced in precision oncology and invites a deeper exploration into the functional dynamics of coexisting oncogenic drivers and their implications for targeted cancer therapies. Future research in this area is vital to unravel these complex molecular interactions and guide effective treatment strategies for patients with similarly unique molecular landscapes.

### Electronic supplementary material

Below is the link to the electronic supplementary material.

Supplementary Material 1

## Data Availability

Not applicable.

## Abbreviations

MEN2: Multiple Endocrine Neoplasia type 2
PTC: Papillary Thyroid Carcinoma
NSCLC: Non-Small-Cell Lung Cancer
CRC: Colorectal Cancer
NCOA4: Nuclear Receptor Coactivator 4
CCDC6: Coiled-Coil Domain Containing 6
KRAS: Kirsten Rat Sarcoma Viral Oncogene Homolog
NRAS: Neuroblastoma RAS Viral Oncogene Homolog
HRAS: Harvey Rat Sarcoma Viral Oncogene Homolog
GTPases: Guanosine Triphosphatases
UICC: Union for International Cancer Control
MSS: Microsatellite Stable
5-FU: 5-fluorouracil
TSO500: TruSight Oncology 500
NGS: Next-Generation Sequencing
IDH1: Isocitrate Dehydrogenase 1
APC: Adenomatous Polyposis Coli
FISH: Fluorescence In Situ Hybridization
EGFR: Epidermal Growth Factor Receptor

## References

[1] Drilon A, Oxnard GR, Tan DSW, Loong HHF, Johnson M, Gainor J (2020). Efficacy of Selpercatinib in RET Fusion–positive non–small-cell Lung Cancer. N Engl J Med.

[2] Subbiah V, Yang D, Velcheti V, Drilon A, Meric-Bernstam F (2020). State-of-the-art strategies for targeting RET-Dependent cancers. J Clin Oncol.

[3] Subbiah V, Wolf J, Konda B, Kang H, Spira A, Weiss J (2022). Tumour-agnostic efficacy and safety of selpercatinib in patients with RET fusion-positive solid tumours other than lung or thyroid tumours (LIBRETTO-001): a phase 1/2, open-label, basket trial. Lancet Oncol.

[4] Kloosterman WP, van den Coebergh RRJ, Pieterse M, van Roosmalen MJ, Sieuwerts AM, Stangl C (2017). A systematic analysis of oncogenic gene fusions in primary Colon cancer. Cancer Res.

[5] Le Rolle A-F, Klempner SJ, Garrett CR, Seery T, Sanford EM, Balasubramanian S (2015). Identification and characterization of RET fusions in advanced colorectal cancer. Oncotarget.

[6] Shi M, Wang W, Zhang J, Li B, Lv D, Wang D (2022). Identification of RET fusions in a Chinese multicancer retrospective analysis by next-generation sequencing. Cancer Sci.

[7] Yaeger R, Chatila WK, Lipsyc MD, Hechtman JF, Cercek A, Sanchez-Vega F (2018). Clinical sequencing defines the genomic Landscape of Metastatic Colorectal Cancer. Cancer Cell.

[8] Pietrantonio F, Di Nicolantonio F, Schrock AB, Lee J, Morano F, Fucà G (2018). RET fusions in a small subset of advanced colorectal cancers at risk of being neglected. Ann Oncol.

[9] Regua AT, Najjar M, Lo H-W (2022). RET signaling pathway and RET inhibitors in human cancer. Front Oncol.

[10] Yang S-R, Aypar U, Rosen EY, Mata DA, Benayed R, Mullaney K (2021). A performance comparison of commonly used assays to detect RET fusions. Clin Cancer Res.

[11] Li S, Balmain A, Counter CM (2018). A model for RAS mutation patterns in cancers: finding the sweet spot. Nat Rev Cancer.

[12] Bailey MH, Tokheim C, Porta-Pardo E, Sengupta S, Bertrand D, Weerasinghe A (2018). Comprehensive characterization of Cancer driver genes and mutations. Cell.

[13] Cook JH, Melloni GEM, Gulhan DC, Park PJ, Haigis KM (2021). The origins and genetic interactions of KRAS mutations are allele- and tissue-specific. Nat Commun.

[14] Strickler JH, Yoshino T, Stevinson K, Eichinger CS, Giannopoulou C, Rehn M (2023). Prevalence of KRAS G12C Mutation and co-mutations and Associated Clinical outcomes in patients with colorectal Cancer: a systematic literature review. Oncologist.

[15] Uhrig S, Ellermann J, Walther T, Burkhardt P, Fröhlich M, Hutter B (2021). Accurate and efficient detection of gene fusions from RNA sequencing data. Genome Res.

[16] Li B, Qu H, Zhang J, Pan W, Liu M, Yan X (2021). Genomic characterization and outcome evaluation of kinome fusions in lung cancer revealed novel druggable fusions. NPJ Precis Oncol.

[17] Meng Y, Li L, Wang H, Chen X, Yue Y, Wang M (2022). Pralsetinib for the treatment of a RET-positive advanced non-small-cell lung cancer patient harboring both ANK-RET and CCDC6-RET fusions with coronary heart disease: a case report. Ann Transl Med.

[18] Urbanska EM, Sørensen JB, Melchior LC, Costa JC, Santoni-Rugiu E (2022). Durable response to combined Osimertinib and Pralsetinib Treatment for Osimertinib Resistance due to Novel intergenic ANK3-RET Fusion in EGFR-Mutated non-small-cell Lung Cancer. JCO Precis Oncol.

[19] Fakih MG, Salvatore L, Esaki T, Modest DP, Lopez-Bravo DP, Taieb J (2023). Sotorasib plus Panitumumab in Refractory Colorectal Cancer with Mutated KRAS G12C. N Engl J Med.

[20] Yaeger R, Weiss J, Pelster MS, Spira AI, Barve M, Ou S-HI (2023). Adagrasib with or without Cetuximab in Colorectal Cancer with Mutated KRAS G12C. N Engl J Med.

[21] Chakravarty D, Gao J, Phillips S, Kundra R, Zhang H, Wang J et al. OncoKB: a Precision Oncology Knowledge Base. JCO Precis Oncol. 2017;:1–16. 10.1200/PO.17.00011 PMC5586540 28890946

[22] Suehnholz SP, Nissan MH, Zhang H, Kundra R, Nandakumar S, Lu C (2023). Quantifying the Expanding Landscape of clinical actionability for patients with Cancer. Cancer Discov.

[23] Gainor JF, Curigliano G, Kim D-W, Lee DH, Besse B, Baik CS (2021). Pralsetinib for RET fusion-positive non-small-cell lung cancer (ARROW): a multi-cohort, open-label, phase 1/2 study. Lancet Oncol.

[24] Wirth LJ, Sherman E, Robinson B, Solomon B, Kang H, Lorch J (2020). Efficacy of Selpercatinib in RET-Altered thyroid cancers. N Engl J Med.

