# Supplementary material for: Concurrent KRAS p.G12C mutation and ANK3::RET fusion in a patient with metastatic colorectal cancer: a case report
Source: Diagn Pathol. 2024 Mar 27;19:55. doi: 10.1186/s13000-024-01478-1 (PMC10976695; doi:10.1186/s13000-024-01478-1)
Supplement: Supplementary file 1 — Supplementary Material 1 [file 13000_2024_1478_MOESM1_ESM.docx]

# SUPPLEMENTAL MATERIAL

## Supplementary Table 1

Gene variants with uncertain significance detected by the TSO500 assay.

| **Gene** | **Allele frequency** | **Coverage** | **Variant (p.)** | **Variant (c.)** | **Exon** | **Information** |
| --- | --- | --- | --- | --- | --- | --- |
| *ARID1B* | 33.6 | 1247 | *NP_001333742.1:*  *p.(Thr2067Met)* | *NM_001346813.1:c.*  *6200C>T* | 20/20 | Known, uncertain significance (ClinVar, gnomAD) |
| *ERCC3* | 56.79 | 1016 | *NP_000113.1:p.(*  *Val177Ile)* | *NM_000122.1:c.529*  *G>A* | 5/15 | Known, uncertain significance (ClinVar, gnomAD) |
| *FAT1* | 47.05 | 950 | *NP_005236.2:p.(L*  *eu4062Arg)* | *NM_005245.3:c.121*  *85T>G* | 23/27 | Known, uncertain significance (ClinVar, gnomAD) |
| *FLT4* | 32.41 | 969 | *NP_891555.2:p.(*  *Gly377Trp)* | *NM_182925.4:c.112*  *9G>T* | 9/30 | Unknown, not found in any of the databases used |
| *GATA2* | 19.73 | 522 | *NP_116027.2:p.(S*  *er277Gly)* | *NM_032638.4:c.829*  *A>G* | 3/6 | Known, uncertain significance (Jax-CKB) |
| *GNAS* | 33.66 | 930 | *NP_536350.2:p.(*  *Glu47Lys)* | *NM_080425.3:c.139*  *G>A* | 1/13 | Known, uncertain significance (gnomAD) |
| *MAP3K4* | 34.42 | 918 | *NP_005913.2:p.(*  *Val413Ile)* | *NM_005922.3:c.123*  *7G>A* | 3/27 | Unknown, not found in any of the databases used |
| *PTPN11* | 21.19 | 656 | *NP_002825.3:p.(Il*  *e310SerfsTer30)* | *NM_002834.3:c.927*  *_931delinsATCATCG* | 8/16 | Significance unknown, truncated protein |
| *PTPRT* | 31.73 | 1100 | *NP_573400.3:p.(P*  *he646Val)* | *NM_133170.3:c.193*  *6T>G* | 12/32 | Unknown, not found in any of the databases used |
| *RASA1* | 83.59 | 512 | *NP_002881.1:p.(T*  *yr480Ser)* | *NM_002890.2:c.143*  *9A>C* | 10/25 | Known, uncertain significance (gnomAD) |
| *SHQ1* | 50.60 | 919 | *NP_060600.2:p.(L*  *ys412Glu)* | *NM_018130.2:c.123*  *4A>G* | 11/11 | Known, uncertain significance (gnomAD) |
| *SLX4* | 35.26 | 916 | *NP_115820.2:p.(*  *Arg237Trp)* | *NM_032444.3:c.709*  *C>T* | 3/15 | Known, uncertain significance (ClinVar, gnomAD) |
| *SMAD2* | 53.71 | 525 | *NP_001003652.1:*  *p.(Thr303Arg)* | *NM_001003652.3:c.*  *908C>G* | 8/11 | Known, uncertain significance, likely pathogenic (ClinVar) |
| *SMAD3* | 53.31 | 529 | *NP_005893.1:p.(T*  *rp232Cys)* | *NM_005902.3:c.696*  *G>C* | 6/9 | Known, uncertain significance (ClinVar, gnomAD) |
| *SOX17* | 37.21 | 301 | *NP_071899.1:p.(*  *Ala279Thr)* | *NM_022454.3:c.835*  *G>A* | 2/2 | Known, uncertain significance (gnomAD) |
| *TBX3* | 46.92 | 471 | *NP_057653.3:p.(P*  *ro135Gln)* | *NM_016569.3:c.404*  *C>A* | 2/8 | Unknown, not found in any of the databases used |
| *TFE3* | 48.58 | 1231 | *NP_006512.2:p.(S*  *er112Leu)* | *NM_006521.5:c.335*  *C>T* | 3/10 | Known, uncertain significance (gnomAD) |

## Supplementary Figure 1


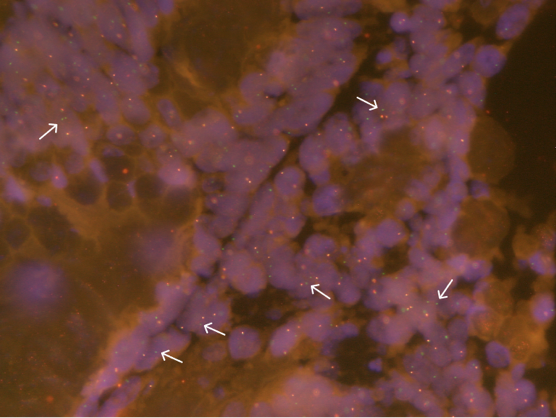


**Fluorescence in situ hybridization (FISH) analysis for *RET* gene rearrangement.** The tissue sample of a tumor-infiltrated lymph node from the initial diagnosis in 2016 was hybridized with a break-apart probe for *RET*, where the separation of red and green signals indicates a translocation involving the *RET* locus. An extra green signal pattern was observed in most of the tumor cells (white arrows) with a few break-apart signals observed as well. Original magnification, x63.
